# School-based obesity prevention interventions in Latin America: A systematic review

**DOI:** 10.11606/s1518-8787.2020054002038

**Published:** 2020-10-28

**Authors:** Rosemary Cosme Chavez, Eun Woo Nam

**Affiliations:** I Yonsei University Yonsei Global Health Center Wonju City Republic of Korea Yonsei University . Yonsei Global Health Center . Wonju City , Republic of Korea; II Yonsei University Graduate School Department of Health Administration Wonju City Republic of Korea Yonsei University . Graduate School . Department of Health Administration . Wonju City , Republic of Korea

**Keywords:** Child, Adolescent, Obesity, prevention & control, Evaluation of the Efficacy-Effectiveness of Interventions, School Health Services, Review

## Abstract

**OBJECTIVE:**

To evaluate the implementation and effectiveness of school-based interventions to prevent obesity conducted in Latin America and provide suggestions for future prevention efforts in countries of the region.

**METHODS:**

Articles published in English, Spanish, and Portuguese between 2000 and 2017 were searched in four online databases (Google Scholar, PubMed, LILACS, and REDALYC). Inclusion criteria were: studies targeting school-aged children and adolescents (6–18 years old), focusing on preventing obesity in a Latin American country using at least one school-based component, reporting at least one obesity-related outcome, comprising controlled or before-and-after design, and including information on intervention components and/or process.

**RESULTS:**

Sixteen studies met the inclusion criteria. Most effective interventions (n = 3) had moderate quality and included multi-component school-based programs to promote health education and parental involvement focused on healthy eating and physical activity behaviors. These studies also presented a better study designs, few limitations for execution, and a minimum duration of six months.

**CONCLUSIONS:**

Evidence-based prevention experiences are important guides for future strategies implemented in the region. Alongside gender differences, an adequate duration, and the combined use of quantitative and qualitative evaluation methods, evidence-based prevention should be considered to provide a clearer and deeper understanding of the true effects of school-based interventions.

## INTRODUCTION

Childhood obesity is a global public health problem. The worldwide trend showed that, in 2013, 22.6% of girls and 23.8% of boys in developed countries were overweight or obese, whereas in developing countries it was 13% for both girls and boys ^1^ . Recently, a study on the trends from 1975 to 2016 estimated the global-age standardized prevalence of obesity in children and adolescents to be 5.6% among girls and 7.8% among boys, highlighting the sustained growth in developing countries ^2^ .

Latin America follows this trend: in 2016, the prevalence of overweight and obesity among 5 to 19 year-old Mexican children and adolescents ranged between 33% (both genders, aged 5–11 years) and 39.2% (girls aged 12–19 years; boys: 33.5%) ^3^ . Country-specific data from the Global Burden of Disease (GBD) 2015 Obesity Collaborators shows that in Chile the prevalence was 25.5% among girls and 36.4% among boys, and in Brazil 23.8% among girls and 27.3% boys (< 20 years old) ^4^ . As for Peru, GBD found 22.4% of girls and 19.2% of boys to be overweight or obese.

Environmental factors, lifestyle preferences, and cultural environments play key roles in the worldwide rising prevalence of obesity ^5^ . Numerous studies showed that sugary beverages and high-fat foods consumption added to low fruit and vegetable intake, decreased physical activity (PA), and increased sedentary behavior is positively associated with obesity ^6^ .

This evinces the importance in promoting healthier food choices and a more active lifestyle along with environment-related changes to improve healthy behavior in school-aged children and adolescents ^9^ . Schools play a pivotal role in promoting a healthy lifestyle among students. Reviews on school-based prevention programs for obesity, mostly in the United States and Europe, reported improvement in health-related behaviors and/or knowledge, as well as some positive impacts on body mass index (BMI) ^9^ .

Reviews on the extent and impact of interventions conducted in Latin America targeting obesity among school-aged children and adolescents are scarce. Most reviews are based on international efforts and include few studies conducted in Latin America, or approach PA interventions and report only PA-related outcomes ^10 , 12^ . A review performed in 2013 reported significant outcomes in seven of the ten included studies, among which at least three presented an appropriate design and execution conducive to statistically significant changes in obesity-related outcomes ^16^ .

Considering that our systematic review seeks to assess how obesity prevention interventions targeting school-aged children and adolescents were implemented in different Latin American countries (i.e. implementation) and whether they were effective on obesity-related outcomes (i.e., effectiveness). Based on these findings, we provide suggestions for future prevention efforts implemented in Latin American countries.

## METHODS

### Literature Search

**Figure 1:**
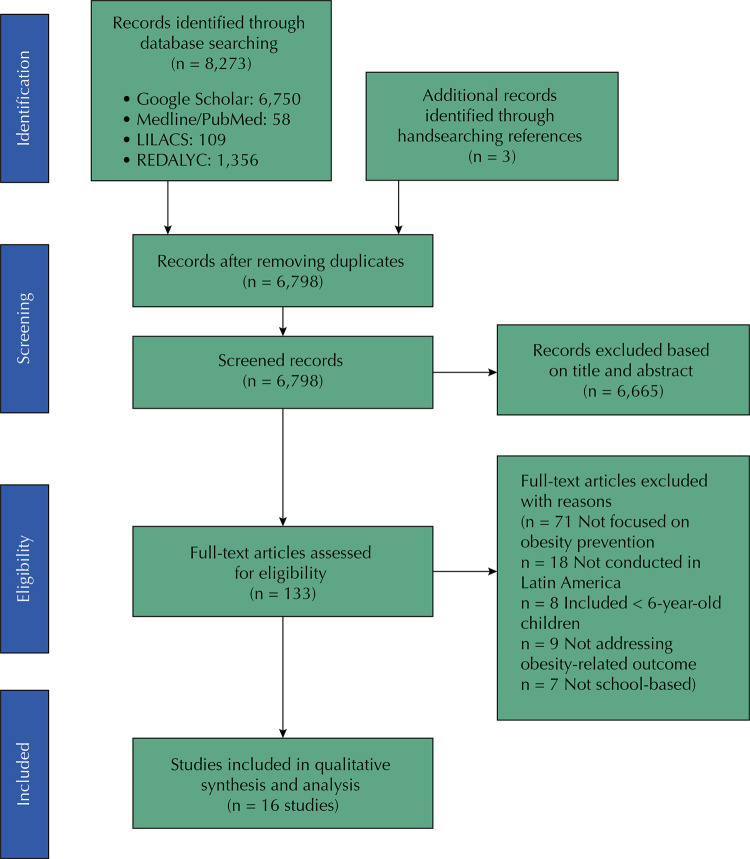
PRISMA literature review flowchart.

Studies published from 2000 to 2017 were collected by searching four online databases: Google Scholar, PubMed, Literature in the Health Sciences in Latin America and the Caribbean (LILACS), and the Network of Scientific Journals of Latin America and the Caribbean, Spain, and Portugal (REDALYC). An update search was conducted in March 2019. Potentially eligible articles were also located by hand, by screening studies and articles reference list, across the 20 Latin American countries. Search strategy included Medical Subject Headings (MeSH) terms combined with text words based on the categories: population, intervention, outcomes, and type of studies addressed by the review. Search strategy is available at https://osf.io/yuz7e/. Publications written in English, Spanish, and Portuguese were covered. Search was conducted in three stages: first, one researcher conducted data search (RCCH); then identified studies were screened based on title and abstract; finally, studies were independently assessed in full-text considering the inclusion/exclusion criteria by two researchers (RCCH and EWN) ( Figure 1 ). In the event of disagreement or conflicts between the researchers, results were discussed based on full-text evaluation until reaching consensus.

### Selection of Studies

Studies were included in this review if they met the following eligibility criteria: a) targeted school-aged children and/or adolescents (6–18 years old) of Latin American countries, b) addressed an intervention for primary prevention of obesity using at least one school-based component, c) reported obesity-related outcomes, d) comprised a controlled or before-and-after design, and e) included information on intervention components and/or process. Exclusion criteria were: a) studies that included children under six years old, categorized as preschool-aged, b) targeted overweight and/or obese children/adolescents as secondary prevention or treatment, c) addressed an intervention that was not conducted in a Latin American country, and d) reported solely dietary and/or PA outcomes.

Studies were not restricted regarding duration, follow-up period, risk of obesity, and intervention implementers.

### Quality Assessment

Study quality was assessed using the standardized Effective Public Health Practice Project Quality Assessment Tool (EPHPP Tool). It comprises six components ^17^: a) selection bias, b) study design, c) confounders, d) blinding, e) data collection methods (validity and reliability of tools), and f) withdrawals or drop-outs per group. Two researchers (RCCH and EWN) independently assessed the collected studies. Following EPHPP guidelines, each criterion was rated as good, fair, or poor, and each study received an overall score by the sum of the six ratings. In the event of any discrepancy between researchers, each study was reassessed collectively to reach a final decision.

### Data Extraction

One researcher (RCCH) extracted details on each study intervention characteristics and summarized them in a table using a narrative synthesis. Collected data consisted of: first author name; publication year; country and specific setting (region-city); intervention focus; number of participants and age; target group (low socioeconomic status [SES]); intervention activities, duration and follow-up; theoretical basis, outcome measures, process and/or cost evaluation; and overall quality evaluation. Outcome measures were considered those related to obesity (prevalence of overweight and/or obesity, BMI, BMI z-score), dietary/nutrition behavior (e.g., fruit, vegetables, fast foods, and snacks intake), and PA behavior (e.g., PA time, exercise tolerance, and endurance). Effectiveness across studies was determined by effect size (ES). The following formula, also adopted by other studies ^18^ , was used to estimate Cohen’s D ES: d = ( *x*_1_ – *x*_2_ )/ *s* , where *x*_1_ is the mean of the intervention group (IG), *x*_2_ the mean of the control group (CG), and *s* the pooled standard deviation. ES was considered trivial if < 0.2, small if equal to 0.2 or < 0.5, medium if equal to 0.5 or < 0.8, and large if equal to or higher than 0.8. For before-and-after study designs, ES was estimated based on the results of the last reported follow-up. ES values were tabulated for each outcome and based on intervention focus (whether dietary/nutrition, PA, or both).

## RESULTS

### Literature Search

Our initial search identified 8,273 publications on the databases and three by handsearching reference lists ( Figure 1 ). After eliminating duplicates and screening studies titles/abstracts, 133 articles remained. By reading articles full-text and applying eligibility criteria, we found 71 articles unrelated to obesity prevention, 18 describing school-based programs not conducted in Latin America, 8 including children under 6 years old (categorized as preschoolers), 9 that did not report obesity-related outcomes, and 7 that were not based on school activities, all of which were excluded. Sixteen interventions (reported in 20 publications) met the inclusion criteria and were included in the systematic review. Several Latin America countries had very few or no school-based studies on neither of the four databases.

### General Characteristics of the Studies

Among the 16 interventions included in our study ^19^ , 5 (31%) were conducted in Mexico ^20 , 25^ , 4 (25%) in Chile ^22 , 23 , 35 , 37^ , 3 in Brazil (19%) ^30^ , 3 in Peru (19%) ^19 , 21 , 34^ , and 1 in Argentina (6%) ^24^ ( Table 1 ). All studies collected data from both genders and most of them were conducted with primary school students ^19 , 21^ (4–14 years old), except one with high school students (14–17 years) ^20^ and one with students from both education levels (6–16 years) ^34^ . Over 30% (n = 5) focused strictly on diet or nutrition ^21 , 24 , 30 , 31 , 36^ , one on PA ^32 , 33^ , and over 60% (n = 10) included both dietary/nutrition and PA intervention programs ^19 , 20 , 22 , 23 , 25 - 29 , 34 , 35 , 37^ . Sample size ranged from 121 to 3,086 subjects, and half of the studies (n = 8) targeted students with low SES ^22^ . Intervention minimum duration was four months and minimum follow-up five, whereas maximum period for both was two years. Only four studies based their intervention design and/or activities on a theoretical framework ^22 , 24 , 25^ , and seven limitedly reported their process (attendance, adherence) ^19 , 21 , 22 , 24^ . A single study reported intervention cost evaluation ^21^ .

**Table 1:** Summary of the characteristics of school-based interventions (n = 16).

First author & year, country, region/city	General characteristics			Intervention					Overall Quality
	Focus	Participants, age (range, average)	Target	Specificities	Duration & follow-up	Theory	Reported outcomes	Process/ Cost evaluation	
Aparco 2017, Peru, Lima ^19^	Diet + PA	696 PS I: 2 schools C: 2 schools, NR, 7.5 y	No	Nutrition and PA education programs for students, education for teachers and parents, PA kits provision, active recess, and leadership formation. Domains: School environment, curriculum, parental involvement	Duration: 9 months Follow-up: 9 months	No known theory	BMI, BMI z-score, WC, food intake, nutrition knowledge, PA level	Adherence Nutrition/PA education: S: 91.6% P: 83.9%	Weak
Elizondo-Montemayor 2014, Mexico ^20^	Diet + PA	304 SS 5 schools, (14-17), NR	No	Lectures, Zumba cardio dance, and sport classes alongside information on healthy eating and PA by website and social networks. Domains: School environment, curriculum	Duration: unclear Follow-up: one school year	No known theory	OV/OB P, BMI, food intake, PA frequency	No	Weak
Gago 2014, Peru, Lima ^21^	Diet	529 PS I: 2 schools C: 2 schools, NR, 7.6 ± SD= 1.9 y	No	Nutrition-education program for students, education for teachers, and practical sessions for parents and teachers per school. Domains: School environment, curriculum, parental involvement	Duration: unclear Follow-up: 11 months	No known theory	OV/OB P, BMI z-score	Attendance T: 95%, P: 55% Av. Cost: USD 20 per student	Weak
Lobos Fernandez 2013, Chile, Santiago ^22^	Diet + PA	796 PS 7 schools, (4-11), 7.6 ± SD= 1.6 y	Yes	Nutrition-education program for students, nutrition training sessions for teachers. PA component, including a weekly increase in PE classes by teachers and sport equipment provision. Domains: School environment, curriculum	Duration: 18 months Follow-up: 2 years	SCT	OV/OB P, BMI, BMI z-score, nutrition knowledge, 6-min walk test	Adherence to Nutrition program: S: 70% Y1, 92.3% Y2 PE classes: 93.6% two year-average	Weak
Ratner 2013, Chile, Santiago ^23^	Diet + PA	2,527 PS, 8 schools, NR, 8.0 (6.9–9.0)	Yes	Nutrition-education program for students and improvement in food environment. PA component, consisting of a PA weekly increase during recess times. Domains: School environment, curriculum	Duration: 2 years Follow-up: 2 years	No known theory	OV/OB P, BMI, BMI z-score	No	Weak
Rausch Herscovici 2013, Argentina, Rosario ^24^	Diet	387 PS I: 4 schools C: 2 schools, (9–11), 9.7 ± SD= 0.73 y	Yes	Nutrition-education program for students, one workshop for parents, and improvement in food environment. Domains: School environment, curriculum	Duration: 6 months Follow-up: 6 months	Social Learning Theory	BMI, BMI z-score, food intake	Attendance P: 53%	Weak
Safdie 2013, Aburto 2011, Mexico, Mexico-City ^25,26^	Diet + PA	886 PS I: 16 schools C: 11 schools, NR, 9.7 ± SD= 0.7 y	Yes	Two intervention intensities: basic and plus. Nutrition-education program for students and improvement in food environment. PA component, including PE classes, PA environment enhancement, and daily exercise session before school day start. Domains: School environment, curriculum	Duration: 18 months Follow-up: 2 years	TPB, SCT, HBM	OV&OB P, BMI, food intake, MVPA, steps per day at school/all day (24 h)	Adherence to Nutrition program: T: 100%	Weak
Bacardí-Gascón 2012, Mexico, Tijuana ^27^	Diet+ PA	532 PS I: 2 schools C: 2 schools, NR, 8.5 ± SD= 0.73 y	No	Nutrition/PA education for students, sessions for school boards and teachers, nutrition-education program for parents, and food environment and PA infrastructure improve. Domains: School environment, curriculum, parental involvement	Duration: 6 months Follow-up: 24 months	Ecological model	OV&OB P, BMI, BMI z-score, WC, food intake, PA time, sedentary behavior	Adherence to Intervention S: > 90%	Moderate
Colin-Ramirez 2010 and Colin-Ramirez 2009, Mexico, Mexico City ^28,29^	Diet + PA	498 PS I: 5 schools C: 5 schools, (8–10), 9.5 ± SD= 0.7 y	Yes	Nutrition-education program for students, suggestions of healthy snacks for vendors, and education/menu suggestions for parents. PA component including PA education, exercise recesses inside the classroom, PE classes, and PA suggestions for parents. Domains: School environment, curriculum, parental involvement.	Duration: 12 months Follow-up: 12 months	No known theory	OV/OB P, MPA/MVPA time, food intake, sedentary behavior	No	Weak
Fernandes 2009, Brazil, Florianopolis ^30^	Diet	135 PS I: 1 school C: 1 school, NR, 8.15 y	No	Nutrition-education program for students on subjects related to healthy diets and snacks, and preparation. Domains: School curriculum	Duration: 4 months Follow-up: 5 months	No known theory	OV/OB P, food intake	No	Weak
Sichieri 2009, Brazil, Rio de Janeiro ^31^	Diet	1134 PS I: 23 classes C: 24 classes, (9–12), 10.9 y	Yes	Healthy lifestyle education program for students on water intake, encouraging its consumption using simple messages. Domains: School curriculum	Duration: 7 months Follow-up: 1 school year	No known theory	OV/OB P, BMI, food intake	No	Weak
Farias et al 2009 and Farias 2008, Brazil, Rondonia ^32,33^	PA	383 PS I: 1 school C: 1 school, (10–15), 12.4 y	No	PA program for students with weekly PE classes divided into 3 sections: 30-min aerobic activity, 30-min playing sports, and 10-min stretching. Domains: School environment	Duration: 9 months Follow-up: 9 months	No known theory	BMI, run endurance, sit-and-reach test	No	Weak
Pérez Villasante 2008, Peru, Ancash ^34^	Diet + PA	121 PS & SS, 1 school, (6–16), 9.6 y	No	Nutrition and PA education program for students, nutrition workshops for parents, one salad festival improvement in food environment, and fortnightly student walking. Domains: School environment, curriculum, parental involvement.	Duration: 5 months Follow-up: 1 year	No known theory	OV/OB P	No	Weak
Kain 2008, Chile, Casablanca, Quillota ^35^	Diet + PA	2,431 PS I: 3 schools C: 1 school, NR, 9.9 y	Yes	I: Nutrition-education program for students, meetings with parents, and community events. PA component consisting of adapted PA program, weekly increase in PA classes, education program for teachers, and family PA events. Due to a lack of resources, Y2 activities were modified. Domains: School environment, curriculum, parental involvement	Duration: 11 months Follow-up: 21 months	No known theory	OB P, BMI, BMI z-score, WC, triceps skinfold, mile test, 20 m shuttle-run test	No	Moderate
Ramirez-Lopez 2005, Mexico, Sonora ^36^	Diet	360 PS I: 254 children C: 106 children, (6–10), 8.5 ± SD= 1.3 y	No	I: A “School Breakfast Program,” which offered a menu of pasteurized milk, cereal, cookie/bread, and juices (average of 468 kcal) consumed 30 min before the start of classes. Domains: None	Duration: 9 months Follow-up: 9 months	No known theory	OV/OB P, BMI, biochemical parameter	No	Weak
Kain 2004, Chile, Santiago, Curicó, Casablanca ^37^	Diet + PA	3,086 PS I: 3 schools C: 2 schools, NR, 10.6 y	Yes	I: Nutrition-education program for students, meetings with parents, improvement in food environment and supporting activities. PA component including weekly increase in PA, daily active recess, adapted PA program, sports equipment provision, and extra activities. Domains: School environment, curriculum, parental involvement	Duration: 6 months Follow-up: 1 school year (9 months)	No known theory	BMI, BMI z-score, WC, triceps skinfold, 20 m shuttle-run test, lower back flexibility	Adherence to Nutrition Program S: 80%, PA: PE Class 100% Attendance P: 50–90%	Moderate

BMI: body mass index; I: intervention; C: control; OV/OB P: overweight and obesity prevalence; S: students; P: parents; PA: physical activity; PE: physical Education; PS: primary-school students; SS: secondary-school students; T: teachers; MPA: moderate PA, MVPA: moderate-to-vigorous PA; WC: waist circumference; NR: not reported; Y1: first year or school year 1; Y2: second year or school year 2, TPB: Theory of Planned Behavior, SCT: Social Cognitive Theory, HBM: Health Belief Model.

### Characteristics of the Intervention

After reviewing each study specificities, we identified three intervention domains, as summarized in Table 1 , following the Health Promoting School Framework developed by the World Health Organization (WHO) ^38^ . The main domains entail school environment, curriculum, and partnership with families and/or the wider community. As most interventions adopted a school curriculum component, only those including at least two more domains were considered multi-component. Most interventions targeting diet alone (four out of five) were mainly educational, promoting classroom sessions encouraging healthy eating ^21 , 24 , 30 , 31^ , whereas the remaining one was a school breakfast program ^36^ . Among these, one was considered multi-component for embracing efforts for environmental change along with education activities for parental involvement ^21^ . The intervention focused on PA compared a curriculum of PA programming with a conventional physical education (PE) class ^32 , 33^ . Interventions targeting nutrition and PA (n = 10) implemented both educational and environmental activities ^20 , 22 , 23 , 25 , 26^ , and six of them were multi-component interventions that also included parental involvement ^19 , 27 , 28 , 29 , 34 , 35 , 37^

### School-Based Studies Outcomes and Effect Sizes

Tables 2–4 show the effect of the interventions on every outcome: first, obesity-related (particularly the prevalence of overweight and/or obesity, BMI, and BMI z-score); and then behavioral outcomes, including PA and diet intake. Over 60% of the collected studies reported a significant effect on at least one dietary/PA or obesity-related outcome ^19^ . Seven studies provided the necessary data for estimating the respective effect size ^20^ , two were randomized controlled trials ^24 , 31^ , three were non-randomized controlled trials ^32 , 33 , 35 , 37^ , two were quasi-experimental studies with a pretest-posttest nonequivalent groups design ^19 , 21^ , and three were before-and-after studies ^20 , 22 , 27^ . Most studies showed a weak overall quality (n = 13) ^19^ , whereas three showed moderate ^27 , 35 , 37^ . Most common limitations were regarding blinding (16 studies), confounders (11 studies), and unclear validity of data collection methods (8 studies).

**Table 2:** Outcome change and effect size in overweight, prevalence of obesity, and BMI for school-based studies reporting a significance level (n = 7).

Overweight and obesity prevalence			Difference effect (%)/ Baseline-adjusted effect			Effect size	Classification	Quality evaluation
Study reference and intervention focus	Study design	(Sub)group	IG pre-test	CG post-test	p-value	Estimate		
Diet only								
Gago 2014 ^21^	Quasi-experimental							Weak
Overweight prevalence		All	-9.5	-1.2	< 0.001			
Obesity prevalence		All	-3.5	+6.1	< 0.001			
Ramirez-Lopez 2005 ^36^	NRCT							Weak
Overweight prevalence		All	28 ± 11	8 ± 7.6	NS	1.98	Large	
Obesity prevalence		All	28 ± 11	9 ± 8.5	NS	1.84	Large	
Diet + PA								
Kain 2008 ^35^	NRCT							Moderate
Obesity prevalence		Girls	-3.8	+0.5	< 0.05			
		Boys	-4.7	-0.2	< 0.05			
BMI and BMI z-score								
Diet only								
Gago 2014 ^21^	Quasi-experimental							Weak
BMI z-score		All	1.05 ± 1.50	1.23 ±1.59	< 0.001	-0.12	Trivial	
Sichieri 2009 ^31^	Cluster RCT							Weak
BMI		All	+0.32	+0.22	NS			
Ramirez-Lopez 2005 ^36^	NRCT							Weak
BMI		All	17.2 ± 0.1	16.9 ± 0.2	NS	2.14	Large	
PA only								
Farias 2008 and Farias et al 2009 ^32,33^	NRCT							Weak
BMI		Girls	19.8 ± 2.5	20.4 ± 3.3	NS	-0.20	Small	
		Boys	20.2 ± 3.2	20.8 ± 3.4	NS	-0.18	Trivial	
Diet + PA								
Rausch-Herscovici 2013 ^24^	RCT							Weak
BMI		Girls	+0.58	+0.56	NS			
		Boys	+0.4	+0.6	NS			
Kain 2008 ^35^	NRCT							Moderate
BMI		Girls	20.1 ± 3.5	20.8 ± 3.8	NS	-0.19	Trivial	
		Boys	19.7 ± 3.2	20.6 ± 3.7	NS	-0.27	Small	
BMI z-score		Girls	0.58 ± 0.9	0.72 ± 0.9	0.003 ^a^	-0.15	Trivial	
		Boys	0.53 ± 0.95	0.72 ± 1.0	< 0.001 ^b^	-0.19	Trivial	
Kain 2004 ^37^	NRCT							Moderate
BMI		Girls	20.0 ± 3.8	19.6 ± 3.8	NS	0.11	Trivial	
		Boys	19.5 ± 3.5	19.2 ± 3.1	< 0.001 ^a^	0.09	Trivial	
BMI z-score		Girls	0.59 ± 0.89	0.40 ± 0.9	NS	0.21	Small	
		Boys	0.51 ± 0.94	0.46 ± 0.81	< 0.001	0.06	Trivial	

BMI: body mass index; IG: intervention group; CG: control group; NRCT: non-randomized controlled trial; RCT: randomized controlled trial; PA: physical activity; NS: non-significant.

^a^ Interaction effect (type x time)

^b^ Interaction effect (type x time x age).

### Obesity-related outcomes

- Prevalence of overweight and/or obesity: few studies reported this outcome (n = 3) ^21 , 35 , 36^ . Two multi-component interventions significantly decreased the prevalence of overweight and/or obesity on -9.5 to -3.5 percentage points ^21 , 35^ (Table 2); no positive ES was reported.
- BMI: seven studies reported changes in BMI, three of which found a statistically significant intervention effect ^21 , 35 , 37^ (Table 2). Among interventions targeting diet alone, one study reported a positive effect on BMI z-score and a trivial ES for the entire sample (-0.12) ^21^ . The intervention targeting PA achieved no significant effect ^32 , 33^ . As for interventions targeting both diet and PA, two studies reported significant effects on BMI or BMI z-score – one for the overall sample (girls -0.15, boys -0.19) ^35^ and one for boys only (BMI 0.09, BMI z-score 0.06) ^37^ . All aforementioned studies comprised multi-component interventions, including parental educational activities on nutrition.

### Behavioral outcomes

- PA: four studies consisted of dietary and PA interventions. The study focused on PA reported and recorded significant effects on physical condition tests and/or PA time ^22 , 27 , 33 , 35 , 37^ (Table 3): all participants improved their performance on two of the three tests reported by Farias (2008), with ES ranging from small to large (0.43-1.22). However, only the boy subgroup improved their performance on the sit and reach test ^33^ . Regarding studies addressing the two interventions, one reported a small positive ES (0.31-0.47) ^27^ on PA time while the others successfully achieved ES ranging from small to medium when reporting on physical condition tests – two of which in the overall sample (0.23 – 0.55) ^22 , 3,7^ and one among boys (ES 0.73) ^35^ .
  **Table 3:** Outcome change and effect size in physical activity for school-based studies reporting a significance level (n = 5).

Study reference and intervention focus	Study design	(Sub)group	IG pre-test	CG post-test	p-value	Estimate	Classification	Quality evaluation
PA-only								
Farias 2008 ^33^	NRCT							Weak
Sit-and-reach test (cm)		Girls	23.6 ± 7.8	25.8 ± 9.2	< 0.05	-0.26	Small	
		Boys	21.9 ± 7.6	21.0 ± 7.7	< 0.05	0.12	Trivial	
Elbow muscle endurance test (number of repetitions)		Girls	7.7 ± 4.8	5.5 ± 5.3	< 0.05	0.43	Small	
		Boys	15.7 ± 9.0	11.8 ± 8.1	< 0.05	0.46	Small	
Long distance run/walk test (m/9 min)		Girls	1126 ± 191	909 ± 166	< 0.05	1.22	Large	
		Boys	1311 ± 258	1118 ± 262	< 0.05	0.74	Medium	
Diet + PA								Weak
Lobos-Fernandez 2013 ^22,a^	Before and After Study							
6-min walk test (mean meters± SD)		All	448 ± 46	458 ± 44	< 0.001	0.23	Small	
Bacardi-Gascon 2012 ^27,a^	Phase 2: Before and After Study							Weak
Physical education time (hours/week)		Phase 2 (All)	0.90 ± 0.39	0.97 ± 0.15	0.003	0.47	Small	
Supervised sports/dance (hours/week)		Phase 2 (All)	1.35 ± 2.01	2.12 ± 2.49	0.0001	0.31	Small	
Kain 2008 ^35^	NRCT							Moderate
Mile test (min)		Girls	4.2 ± 2.33	4.2 ± 2.35	0.005 ^b^	0.00	Trivial	
		Boys	4.16 ± 2.28	4.17 ± 1.93	0.037 ^b^	0.00	Trivial	
20 m shuttle-run test (stages)		Girls	3.05 ± 1.2	2.49 ± 1.1	0.0007 ^b^	0.48	Small	
		Boys	4.95 ± 1.8	3.65 ± 1.7	< 0.0001 ^b^	0.73	Medium	
Kain 2004 ^37^	NRCT							Moderate
Lower back flexibility test (cm)		Girls	25.7 ± 7.9	23 ± 6.4	< 0.0001 ^b^	0.36	Small	
		Boys	23.6 ± 8.6	22 ± 6.3	< 0.001 ^b^	0.20	Small	
20 m shuttle-run test (stages)		Girls	3.3 ± 1.4	2.6 ± 1.3	< 0.0001 ^b^	0.51	Medium	
		Boys	5.0 ±1.9	3.96 ±1.9	< 0.001 ^b^	0.55	Medium	

IG: intervention group; CG: control group; NRCT: non-randomized controlled trial; PA: physical activity; SD: standard deviation.

^a^ For before-and-after study designs, effect sizes were estimated based on the last follow-up outcome reported.

^b^ Interaction effect (type x time).
- Diet: five studies recorded positive effects on one or several dietary behaviors (two targeting diet-only and three diet and PA) ^19 , 20 , 24 , 27 , 31^ . However, we managed to estimate ES for only two of the interventions targeting both domains (Table 4). One intervention targeting diet alone reported a one-percentage point increase in the daily intake of two healthy food (orange juice and skim milk) among girls and a significant decrease in the consumption of hamburger and hotdog among both boys and girls ^24^ . As for interventions targeting both domains, one managed to sustain fruits intake in the IG (≥ 5 days/week), but reported a significant decrease in the CG (-10.3%). Whereas IG also showed an increasing water intake (+6.1%), in CG it decreased similarly to fruits (-10.6%) ^19^ . The two remaining studies reported a trivial and a small positive ES on similar behaviors (ranging from 0.11 to 0.29) ^20 , 27^ .
  **Table 4:** Outcome change and effect size in diet or nutrition for school-based studies reporting a significance level (n = 5).

Study reference and intervention focus	Study design	(Sub)group	Difference effect (%)/ Baseline-adjusted effect			Effect size		Quality evaluation
			IG pre-test	CG post-test	p-value	Estimate	Classification	
Diet-only								
Rausch Herscovici 2013 ^24^	RCT							Weak
Orange juice intake (%children ≥ 1/day)		Girls	+32.7	+25.6	0.05			
		Boys	+18.9	+21.2	NS			
Skim milk intake (≥ 1/day)		Girls	+27.4	+16.7	0.03			
		Boys	+13.9	+15.0	NS			
Hamburgers/Hot Dogs intake (> 2–4 times/week)		Girls	-38.3	-34.6	> 0.001			
		Boys	-34.7	-26.7	0.01			
Sichieri 2009 ^31^	Cluster RCT							Weak
Sugar-sweetened carbonated beverage intake (per class in mL/d)		All	-69	-13	< 0.05			
Diet + PA								
Aparco 2017 ^19^	Quasi-experimental							
Fruit intake (≥ 5 days/week)		All	+2.0	-10.3	0.004			Weak
Water intake (≥ 5 days/week)		All	+6.1	-10.6	0.019			
Elizondo-Montemayor 2014 ^20,a^	Before and After Study							Weak
Fruit intake (in portions/day)		All	1.9 ± 1.3	2.3 ± 1.4	0.00	0.29	Small	
Vegetable intake (in portions/day)		All	2.4 ± 1.6	2.8 ± 1.6	0.00	0.25	Small	
Bacardi-Gascon 2012 ^27,a^	Phase 2: Before and After Study							Weak
Vegetable intake (in portions/day)		Phase 2 (All)	0.43 ± 0.59	0.52 ± 0.60	0.007	0.15	Trivial	
Snacks containing fat and salt (in portions/day)		Phase 2 (All)	0.33 ± 0.84	0.25 ± 0.70	0.03	0.11	Trivial	

IG: intervention group; CG: control group; RCT: randomized controlled trial; PA: physical activity; NS: non-significant.

^a^ For before-and-after study designs, effect size was estimated based on the last reported follow-up outcome.

## DISCUSSION

Among the 16 studies that met inclusion criteria and were included in our systematic review, 10 (60%) achieved significant positive effects on at least one of their reported outcomes. Of these, three interventions focused on diet, one on PA, and six on both PA and diet. Within the three diet-only interventions, two reported an improvement in dietary behaviors, but failed in positively impacting an obesity-related outcome ^24 , 31^ , and one reduced both of its reported obesity-related outcomes (prevalence of overweight and obesity) by reducing BMI z-score in IG ^21^ . The PA-only intervention managed to improve PA performance within the overall sample for most tests, but this effect had no significantly impact on any obesity-related outcome ^33^ . Of the six diet/PA-focused interventions, three managed to improve a single behavioral outcome ^19 , 20 , 22^ , one positively affected both diet and PA, and two impacted both behavioral and obesity-related outcomes (one PA+ BMI; one PA+ BMI+ obesity prevalence) ^35 , 37^ .

Considering these results, we identified evidence-based effectiveness in three obesity prevention interventions conducted in Latin America that targeted promoting healthy diet and PA by associating environmental, educational, and parental involvement activities (multi-component), corroborating other international reviews ^12 , 39 , 40^ . Although these interventions ES often range from trivial to small, they have a significant capability of providing benefits if scaled to a greater level.

The results of two interventions implemented in Chile and one in Mexico were strengthened based on these studies, showing the best methodological quality (moderate) among our sample ^27 , 35 , 37^ . However, this data also stress the weakness within this research field in Latin America – as already reported by prior studies ^41 , 42^ – regarding the amount and methodological quality of publications. Our systematic review found studies conducted in only 5 of the 20 countries in the region (25% representation) to meet inclusion criteria, and most of them presented a poor methodological quality.

Parental involvement has been considered of key influence for improving children’s lifestyle behaviors and preventing obesity. Our findings emphasize the importance of their role within the school setting, corroborating other reviews worldwide ^10 , 43^ , which may also be explained by the culture of family tradition in Latin America ^10^ .

The potential influence of age in intervention effects is an ongoing debate. While some studies argue that programs targeting older students tend to achieve better outcomes ^11 , 44^ , other meta-analysis found younger children (elementary school students, aged 4–9 years) to experience greater BMI effects than middle (10–13 years) and high school students (14 years or older) ^45^ . However, regional and national disparities in education systems should be regarded when comparing and interpreting results based on age. In Latin America, the education system is characterized by three basic levels: pre-school, primary school, and high school, which, in most countries, entails students aged approximately 3–5 years, 6–13 years, and 13–18 years ^46 , 47^ , respectively. In our review, thirteen studies targeted primary school students, ranging from 7.6 to 10.9 years old (an average of 9.09 years). We suggest further studies to approach both children and adolescents, enabling a deeper understanding regarding age impact on intervention outcomes.

The greater improvement on boys’ physical condition after interventions, reported in two studies, were previously discussed ^48 , 49^ . Considering these findings, it seems that boys tend to engage in more vigorous activity than girls, who tend to be less active ^9^ . One study found a greater improvement in dietary intake among girls ^24^ . Such differences may be explained by the intervention focus, which did not address PA specifically, but rather educational components grounded on social learning theory, to which Kropski et al. (2008) suggest girls might respond better. Yet, further research are required for a better discernment of gender differences within PA and dietary intake behaviors.

Intervention duration and its association with effectiveness are still an ongoing debate, as well as an agreed differentiation cut-off. Bautista-Castano et al. (2004) found that interventions lasting between 6 months and one year are more effective (triceps skin-fold and BMI anthropometrics) than shorter and longer-term interventions ^50^ . Another meta-analysis found short-term interventions (0 to 12 weeks) to have negative effects on BMI, whereas longer interventions (13 weeks or more) are associated with small, significant, and positive BMI effects ^45^ . The duration of the three effective interventions identified in our systematic review ranged from 6 to 11 months, within the cutoff suggested by the aforementioned reviews. Besides duration, follow-up period may also be important for identifying intervention sustained benefits. However, these data are often disregarded in the literature, which might be justified by the difficulty in assessing effects of groups no longer under the intervention arm.

Around 40% of the studies reported evaluation process mostly regarding the overall percentage of individuals targeted by the intervention (students, parents, and teachers) who participated or attended nutrition or PA sessions, that is: the percentages of adherence or attendance. Yet, various researchers argue the need of a throughout evaluation of the implementation process to better contextualize and assess the program true effect ^51 , 52^ . Such need is even sharper in complex or multi-component interventions ^53^ that are not solely based on education. Considering that, we recommend further study to employ both qualitative and quantitative approaches by a mixed-method design, to better plan, correct, and evaluate interventions and their affecting factors.

Our systematic review pose some limitations. First, comparing and interpreting effect sizes from heterogeneous studies targeting children and adolescents is always challenging, and only a few studies provided the appropriate pediatric measures of obesity-related outcomes (BMI z-scores). Second, many methodological deficiencies identified by the quality assessment instrument owed to lack of information, so we urge studies to provide more detailed information regarding the adopted methods. Third, we considered intervention effects regardless of the evaluation data process, because these results were not thoughtfully reported. As our focus was obesity-related outcomes, we might have potentially excluded studies addressing nutrition and PA behaviors as primary outcomes that also contributed to obesity prevention, not only by weight loss.

## CONCLUSIONS

We found evidence of the effectiveness of three school-based interventions for preventing obesity among school-aged children and adolescents in Latin America. These interventions were characterized as moderate quality, included the multi-components of health education, school environment, and parental involvement focusing on healthy eating and PA behaviors, and had better study designs, few execution limitations, and a 6-month minimum duration. Future efforts on preventing obesity in Latin American countries should consider evidence-based preventions experiences, such as those identified in our review, as guides. They should also consider gender differences, appropriate duration, and mixed-method evaluation designs combining both quantitative and qualitative approaches, as their association could provide a clearer and deeper understanding of the school-based interventions true effect.
